# Control of Mitochondrial Morphology Through Differential Interactions of Mitochondrial Fusion and Fission Proteins

**DOI:** 10.1371/journal.pone.0020655

**Published:** 2011-05-27

**Authors:** Pinwei Huang, Chad A. Galloway, Yisang Yoon

**Affiliations:** Mitochondrial Research and Innovation Group, Department of Anesthesiology, Department of Pharmacology and Physiology, University of Rochester School of Medicine and Dentistry, Rochester, New York, United States of America; Centre National de la Recherche Scientifique, France

## Abstract

Mitochondria in mammals are organized into tubular networks that undergo frequent shape change. Mitochondrial fission and fusion are the main components mediating the mitochondrial shape change. Perturbation of the fission/fusion balance is associated with many disease conditions. However, underlying mechanisms of the fission/fusion balance are not well understood. Mitochondrial fission in mammals requires the dynamin-like protein DLP1/Drp1 that is recruited to the mitochondrial surface, possibly through the membrane-anchored protein Fis1 or Mff. Additional dynamin-related GTPases, mitofusin (Mfn) and OPA1, are associated with the outer and inner mitochondrial membranes, respectively, and mediate fusion of the respective membranes. In this study, we found that two heptad-repeat regions (HR1 and HR2) of Mfn2 interact with each other, and that Mfn2 also interacts with the fission protein DLP1. The association of the two heptad-repeats of Mfn2 is fusion inhibitory whereas a positive role of the Mfn2/DLP1 interaction in mitochondrial fusion is suggested. Our results imply that the differential binding of Mfn2-HR1 to HR2 and DLP1 regulates mitochondrial fusion and that DLP1 may act as a regulatory factor for efficient execution of both fusion and fission of mitochondria.

## Introduction

Mitochondria in many cell types are organized into reticular networks composed of filamentous tubules that frequently change shape. Mitochondrial shape change is mediated mainly by fission and fusion of mitochondrial tubules [1]. Multiple proteins have been identified to mediate mitochondrial fission and fusion processes [2]. The most notable proteins are the dynamin family of large GTPases that remodel mitochondrial membranes during the fission and fusion events. The dynamin-like protein DLP1/Drp1 in mammals resides mostly in the cytosol and becomes associated to the outer surface of mitochondria where it is presumed to squeeze and sever the membrane tubule for fission [3]–[5]. The small helix-rich mitochondrial outer membrane protein Fis1 is likely to function as a receptor for DLP1 recruitment to mitochondria [6]–[9]. A recent report suggests that a tail-anchored mitochondrial outer membrane protein, mitochondrial fission factor (Mff), may also function as a DLP1-recruting molecule [10]. Additional dynamin-related GTPases, Mitofusin (Mfn) and optic atrophy 1 (OPA1) are associated with the mitochondrial outer and inner membranes, respectively, and mediate fusion of respective membranes [11]–[16].

Under normal conditions, mitochondrial fission and fusion occur in a balanced frequency and thus relatively constant tubular morphology is maintained. However, perturbation of the fission/fusion balance causes mitochondrial deformation and has been found to be associated with numerous human diseases [17], [18]. Defects in proteins involved in mitochondrial dynamics cause Charcot-Marie-Tooth disease and autosomal dominant optic atrophy [19]–[22]. In addition, genetic dysregulation of the mitochondrial fusion protein Mfn2 has been associated with vascular proliferative disorders [23], as well as obesity and type 2 diabetes [24]. While the functional significance of mitochondrial fission and fusion is evident, the molecular mechanisms of how mitochondrial fission and fusion are regulated are not fully understood.

**Figure 1:**
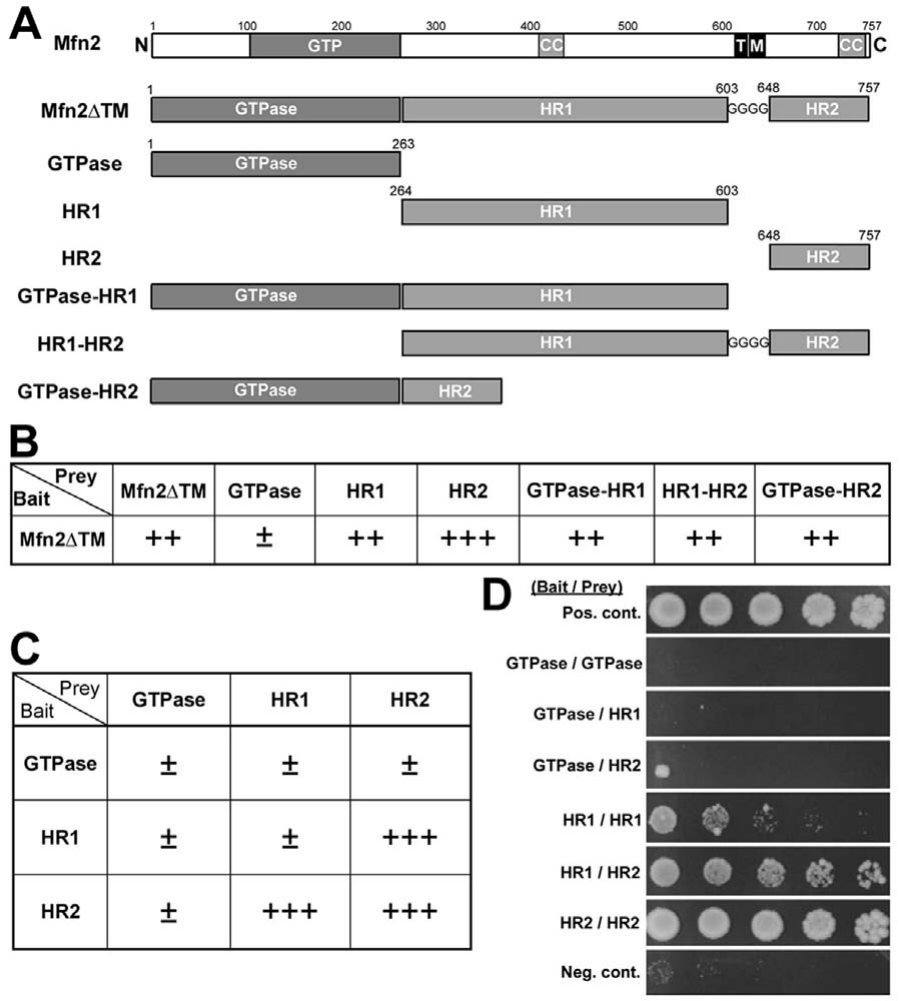
Mfn2 HR1 interacts with HR2. (A) Mfn2 constructs used in Y2H analyses. The transmembrane region is substituted with polyglycine (Mfn2ΔTM) to avoid the protein mislocalization. (B) Semi-quantitative Y2H assays using Mfn2ΔTM as bait and different Mfn2 domains as prey. HIS3 activity represented the strength of the bait-prey interaction and was assessed by percentage of colonies growing on histidine-lacking medium: >75%, ++++; 50–75%, +++; 25–50%, ++; 0–25%, ±. Prey constructs containing HR1, HR2, or both interact with Mfn2ΔTM. (C and D) Y2H assays using individual Mfn2 domains as bait and prey. Both semi-quantitative (C) and qualitative dilution series assays (D) show an interaction between HR1 and HR2 in addition to the HR2/HR2 interaction.

Mammalian cells have two mitofusin isoforms, Mfn1 and Mfn2 [25]–[28]. Both isoforms share a conserved molecular structure containing the N-terminal GTPase domain, two regions of hydrophobic heptad-repeat coiled-coil motifs, HR1 and HR2, and two tandem transmembrane domains near the C-terminus [11], [29] (Fig. 1A). HR1- and HR2-containing regions lie at opposite sides of the bipartite transmembrane domain that spans the outer membrane twice, exposing the GTPase, HR1, and HR2 domains to the cytosol. Studies using Mfn1 showed that the C-terminal HR2 region interacts with the HR2 of another molecule [11]. The HR2/HR2 interaction has been shown to occur in an anti-parallel manner, which allows tethering of the two membranes for subsequent fusion of outer mitochondrial membranes [11].

Intra- and inter-molecular interactions play an important role in membrane remodeling action of dynamin family proteins [30]–[34]. In this study, we examined molecular interactions of the mitochondrial fusion protein Mfn2. We identified an interaction between HR1 and HR2 domains of Mfn2 and found that the association of the two heptad-repeats of Mfn2 is inhibitory of fusion. Additionally, we discovered a previously unidentified interaction between HR1 of Mfn2 and the C-terminal coiled-coil domain of the fission protein DLP1, which was implicated in promoting mitochondrial fusion. Similar to the previous report showing a membrane-fission dynamin participating in yeast vacuole fusion through interaction with a fusion t-SNARE [35], our data demonstrate that the physical interaction between fission and fusion proteins provides a novel mechanism for regulating mitochondrial morphology.

## Results

### The N-terminal HR1 of Mfn2 interacts with the C-terminal HR2 through the predicted coiled-coil region

**Figure 2:**
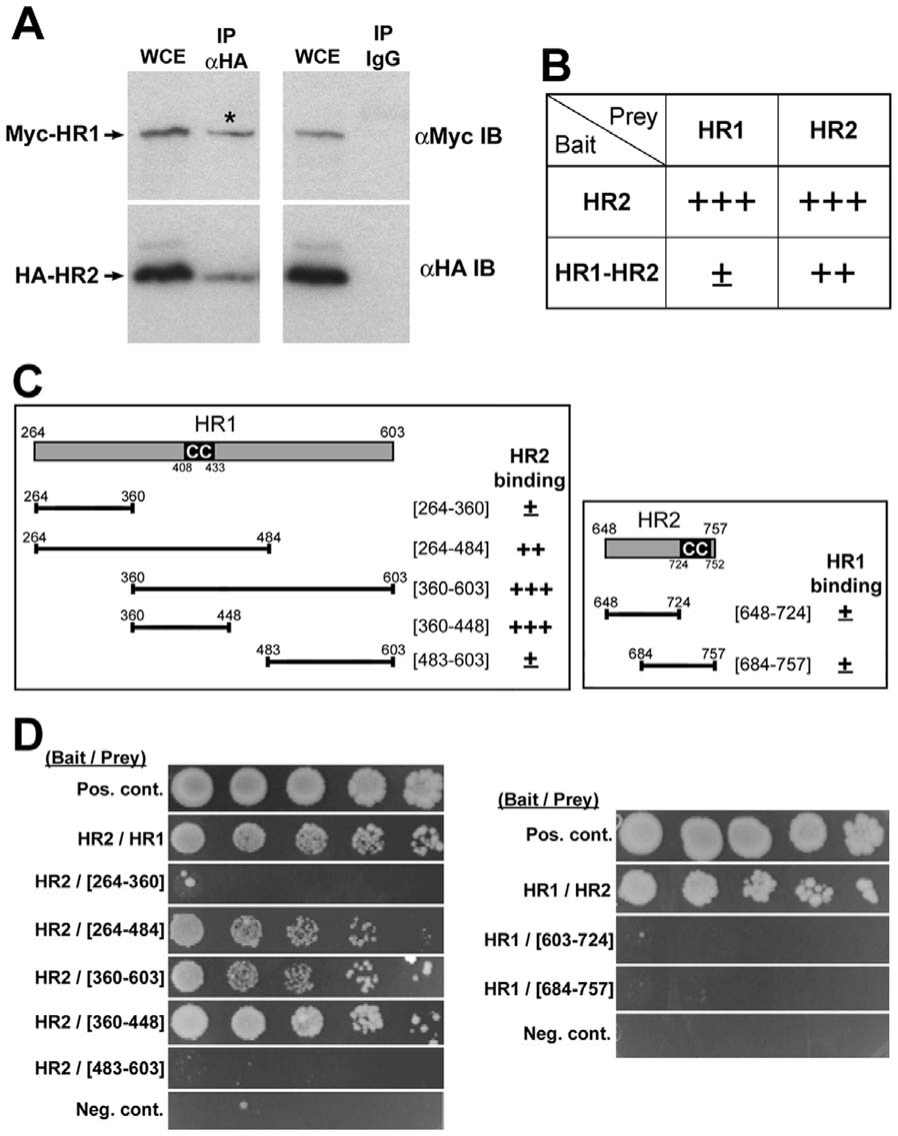
Characterization of the Mfn2-HR1/HR2 interaction. (A) Co-immunoprecipitation of Mfn2-HR1 and HR2. Whole cell extract (WCE) from BHK-21 cells co-transfected with Myc-tagged HR1 and HA-tagged HR2 was subjected to the anti-HA immunoprecipitation. Anti-Myc immunoblotting shows the co-precipitation of Myc-HR1 (asterisk) with HA-HR2. Rabbit IgG was used as a negative control. (B) Association of HR1 and HR2 within bait molecules. Y2H assays using bait containing both HR1 and HR2 in the same molecule (HR1-HR2) show a drastic decrease of HR1 (prey) binding compared to the HR2-only bait, indicating that HR1 and HR2 are preoccupied in bait molecules. The use of HR2 as prey displayed a reduced binding as well although the extent of the decrease was not as great. (C and D) Y2H assays to narrow down the interaction regions within the HR1 and HR2. Semi-quantitative (C) and dilution series (D) analyses demonstrate that Mfn2[360–448] interacts with the HR2 region.

We examined the molecular interactions of Mfn2 using yeast two-hybrid (Y2H) analyses. The domains tested in Y2H analyses include GTPase, HR1, HR2, GTPase-HR1, GTPase-HR2, HR1-HR2, and full-length Mfn2 (Fig. 1A). Throughout this manuscript, we refer to the N-terminal extension and GTPase domain as “GTPase”, the rest of the N-terminal cytosolic domain as “HR1”, and the HR2-containing C-terminal cytosolic domain as “HR2” (Fig. 1A). For the full-length and HR1-HR2 constructs, we replaced the transmembrane domain with polyglycine to avoid mislocalization of the expressed proteins in Y2H analyses. In the initial analyses using the full-length Mfn2 (Mfn2ΔTM) as bait and individual domains as prey, we found that constructs containing HR1, HR2, or both, interact with Mfn2ΔTM (Fig. 1B). While these results were consistent with a previous report that showed an interaction between the two HR2 domains of Mfn1 [11], our data indicate that there is an interaction involving the HR1 domain of Mfn2. The subsequent Y2H assays using individual GTPase, HR1, and HR2 regions as both bait and prey showed that HR1 interacts with HR2 but little with HR1, whereas HR2 interacts well with both HR1 and HR2 (Fig. 1C). Dilution series analyses also revealed that HR1 interacts with HR2 (Fig. 1D). The HR1/HR2 interaction appeared less strong compared to the HR2/HR2 interaction in this assay. Although we occasionally detected a limited growth indicating a potential interaction between two HR1 domains, this interaction is currently inconclusive. We next epitope-tagged HR1 and HR2 with Myc and HA, respectively, and performed co-immunoprecipitation in cultured cells co-transfected with both constructs. As shown in Fig. 2A, Myc-HR1 was detected in the anti-HA immune complex along with HA-HR2, indicating that the two domains interact with each other. This result supports the Y2H data and indicates that the HR1/HR2 interaction occurs in the cellular context.

In Y2H analyses using HR2 alone or HR1-HR2 as bait, and either HR1 or HR2 as prey, we found that the HR1 binding to bait containing both HR1 and HR2 in the same molecule was markedly reduced (Fig. 2B). HR2 showed reduced binding to this bait molecule as well, but the decrease was less pronounced compared to the HR1 binding. Because HR1 can interact with HR2, these results suggest that HR1 and HR2 are associated within the bait molecule. In additional GST pull-down experiments, when GST-HR2 was incubated with the mixture of both HR1 and HR1-HR2, we found that more HR1 bound to GST-HR2 than HR1-HR2, suggesting that the presence of HR1 and HR2 in the same molecule decreases its binding to HR2 (supplemental Fig. S1A). While the configuration of homomeric complexes of Mfn2 is currently unclear, it is possible that the HR1/HR2 interaction occurs either intra-molecularly within the same Mfn2 molecule, or inter-molecularly to form a dimeric or multimeric configuration of Mfn2 proteins (Supplemental Fig. S1B).

To further narrow down the region responsible for the HR1/HR2 interaction, HR2 bindings to different regions of HR1 were tested. Both semi-quantitative and dilution series Y2H assays demonstrated that Mfn2[360–448] binds to the HR2 domain (Fig. 2C and D). We also tested for the HR2 region, but any further deletions within the HR2 abolished the HR1 binding, indicating that the full HR2 region is necessary for the interaction with HR1 (Fig. 2C and D). Our data demonstrate that Mfn2[360–448] that contains the HR1 coiled-coil motif interacts with HR2.

### Mutations in the binding region of Mfn2-HR1 differentially affect mitochondrial morphology

**Figure 3:**
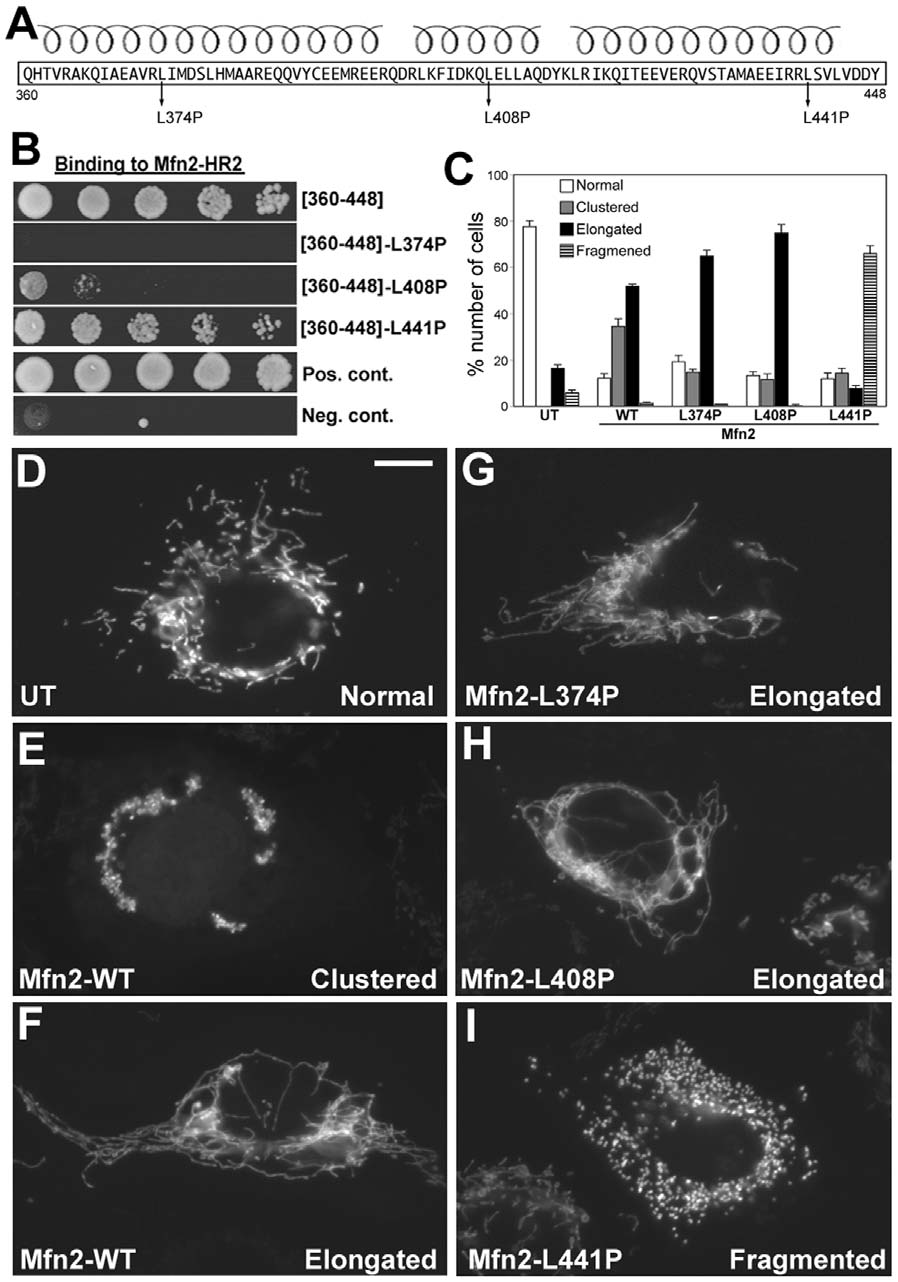
Differential binding effects of three HR1 helix-breaking mutation. (A) Three leucine-to-proline mutations (L374P, L408P, and L441P) made in each of the three predicted helices in the Mfn2[360–448]. (B) Y2H assays testing interactions of three HR1 mutants with Mfn2-HR2. The Mfn2[360–448]-L374P and L408P disrupted the HR2 interaction whereas the L441P mutation maintained it. (C–H) Differential effects of three HR1 helix-breaking mutants on mitochondrial morphology. Clone 9 cells containing GFP-labeled mitochondria were transfected with Myc-tagged wild type Mfn2, Mfn2-L374P, Mfn2-L408P, or Mfn2-L441P, and mitochondrial morphology was evaluated in transfected cells that were identified by anti-Myc staining. (C) Cell counting for different mitochondrial morphologies among transfected cells. UT: untransfected. Error bars represent SEM. While normal tubular mitochondria were seen in untransfected cells (D), overexpression of wild-type Mfn2 induced mitochondrial clustering (E) as well as elongation of mitochondrial tubules (F). Mitochondria were overly elongated and entangled in the majority of cells overexpressing Mfn2-L374P (G) or Mfn2-L408P (H). Cells overexpressing Mfn2-L441P contained fragmented mitochondria (I). Scale bar: 10 µm.

Mfn2[360–448], which we identified as the region binding to Mfn2-HR2, is predicted to contain three α-helical structures that are potentially involved in protein interactions (Fig. 3A). To test the role of these helices in interactions with Mfn2-HR2, we generated three helix-breaking leucine-to-proline mutations, L374P, L408P, and L441P, in each helix of Mfn2-HR1[360–448] (Fig. 3A). Binding analyses by Y2H show that L374P and L408P mutations disrupt the interactions with HR2 whereas the L441P mutation maintains the HR1/HR2 interaction (Fig. 3B). Using these HR1 mutants that have selective binding characteristics to HR2, we tested their effects on the overall morphology of mitochondria. Individual Myc-tagged full-length Mfn2 mutants were expressed in cells for morphological analyses. Overexpression of wild-type Mfn2 induced mitochondrial elongation in addition to forming mitochondrial clusters as previously reported [25], [36], [37] (Fig. 3E, F). Cells transfected with the Mfn2-L374P or Mfn2-L408P mutant showed mitochondrial phenotypes similar to those with the wild-type Mfn2. A large number of cells displayed overly elongated and entangled mitochondrial tubules (Fig. 3G, H). These mutants showed an increased number of cells containing elongated mitochondria compared to wild-type Mfn2 (Fig. 3C), suggesting that abolishing the HR1/HR2 interaction might enhance mitochondrial fusion. A completely opposite phenotype was observed in cells overexpressing Mfn2 containing the L441P mutation that maintains the HR1/HR2 interaction. These cells contained finely fragmented mitochondria (Fig. 3I), indicating a dominant-negative effect of the Mfn2-L441P mutant. Because this mutation maintains the HR1/HR2 interaction, this result suggests that the HR1/HR2 interaction has an inhibitory role in mitochondrial fusion. Computer-assisted morphometric analyses of mitochondrial morphologies demonstrate that mitochondria in cells expressing Mfn2-WT, L374P or L408P have increased values of both aspect ratio and form factor compared to untransfected cells. However, those cells expressing Mfn2-L441P show the value close to 1 for small circular morphology (Supplemental Fig. S2). In addition, the number of mitochondria increased in the Mfn2-L441P mutant-expressing cells while decreasing with the expression of the other mutants and wild type Mfn2, indicating mitochondrial fragmentation and the increased fusion, respectively (Supplemental Fig. S2).

### Mfn2 with the HR1/HR2 association is incompetent for mitochondrial fusion

**Figure 4:**
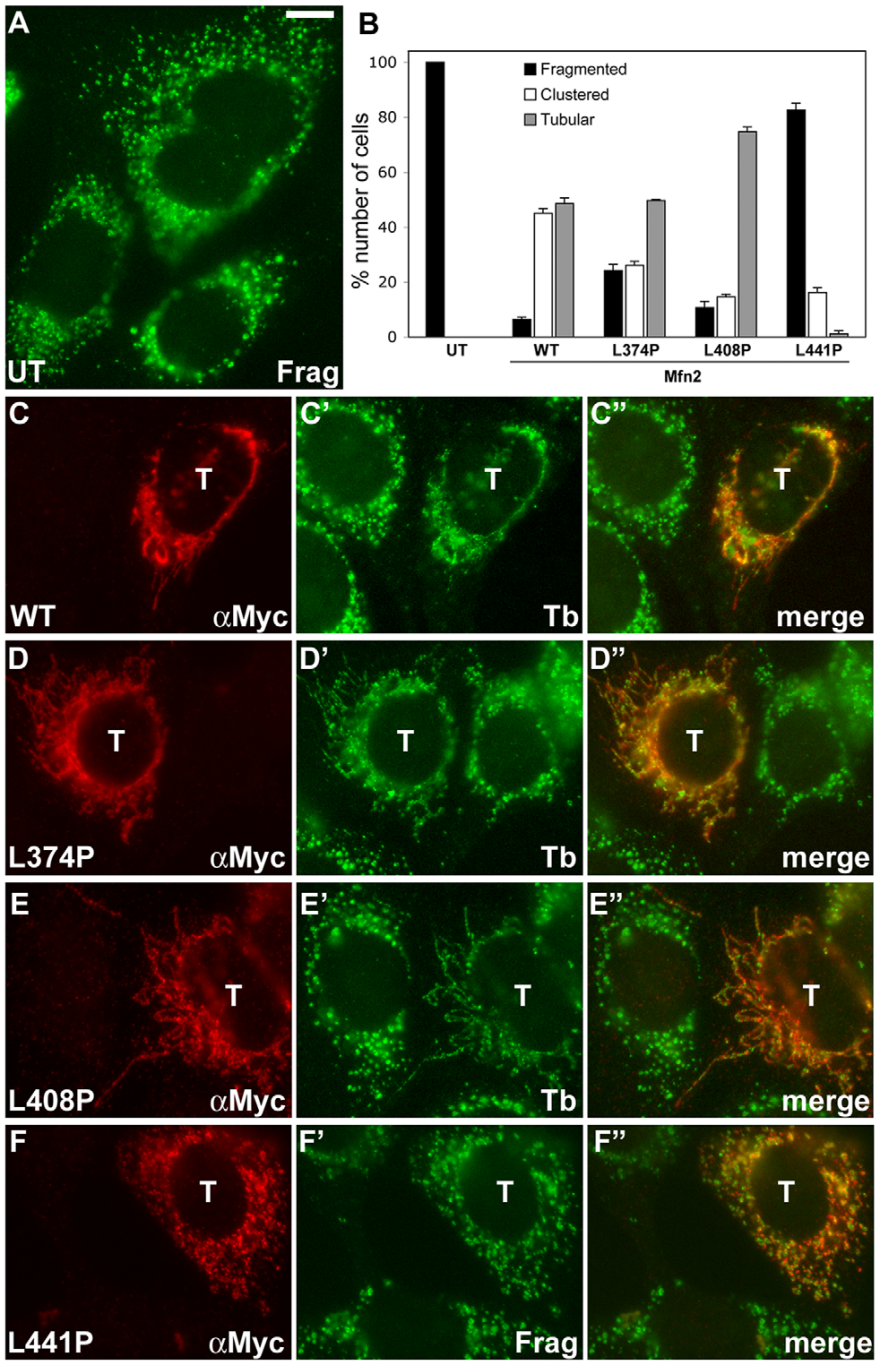
Fusion competency of three Mfn2 mutants. Mfn DKO cells were transfected with Myc-tagged wild type or the three Mfn2 mutants, and co-stained for Myc (red) and mitochondria (green). (A) Mitochondria in untransfected Mfn DKO cells are completely fragmented. UT: untransfected. (B) Mitochondrial morphology quantification. Mfn2-L374P and Mfn2-L408P restored tubular mitochondria similar to or more effectively than wild-type Mfn2. The majority of cells transfected with Mfn2-L441P still contained fragmented mitochondria, indicating that Mfn2-L441P is fusion incompetent. Error bars represent SEM. (C–C”) Mfn DKO cells expressing wild-type Mfn2. The transfected cell positive for Myc staining (C) contains tubular mitochondria (C'). (D–D”, E–E”) Mfn DKO cells transfected with Mfn2-L374P (D) and Mfn2-L408P (E) show tubular mitochondria (D', E'). (F–F”) Mitochondria in the Mfn DKO cell expressing Mfn2-L441P (F) remained fragmented (F'). T: transfected cells. Scale bar: 10 µm.

To specifically address the mitochondrial fusion capacity affected by these Mfn2 mutations, we used Mfn double knockout (DKO) mouse embryonic fibroblasts [11]. Mfn DKO cells contain fragmented mitochondria due to a lack of mitochondrial fusion (Fig. 4A), and expression of wild-type Mfn2 in these cells restores the fusion activity and forms tubular mitochondria [11] (Fig. 4B and C–C”). It was noted that tightly clustered mitochondria were still formed in about a half of the Mfn DKO cells transfected with Mfn2 (Fig. 4B). Next, we expressed individual mutants in Mfn DKO cells and examined mitochondrial morphology. We found that expression of Mfn2-L374P and Mfn2-L408P restored the tubular mitochondria in Mfn DKO cells (Fig. 4B, D–D”, E–E”), indicating that these mutant Mfn2 proteins are capable of mediating mitochondrial fusion. Because both mutations disrupt the Mfn2-HR1/HR2 interaction, this result suggests that the HR1/HR2 interaction is not necessary for mitochondrial fusion. On the other hand, the Mfn2-L441P mutation that maintains the HR1/HR2 interaction did not restore tubular mitochondria in Mfn DKO cells (Fig. 4B, F–F”). The majority of cells transfected with Mfn2-L441P still contained completely fragmented mitochondria, indicating that this Mfn2 mutant is fusion incompetent (Fig. 4B). The fusion incompetence caused by the L441P mutation suggests that dissociating the HR1/HR2 interaction is necessary for mitochondrial fusion.

### Overexpression of Mfn2-HR1 elongates mitochondrial tubules

**Figure 5:**
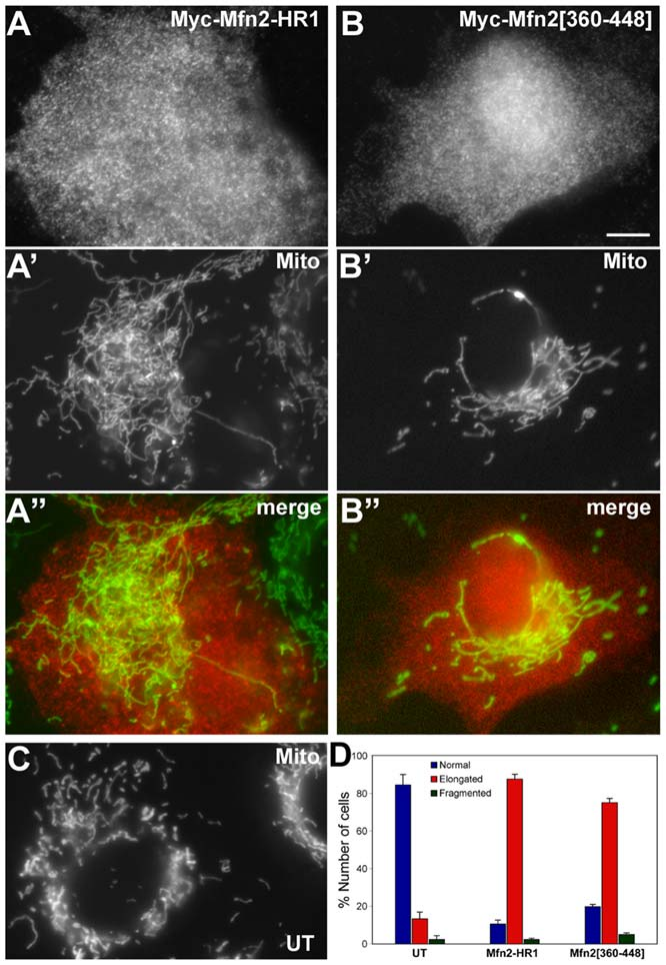
Overexpression of Mfn2-HR1 or Mfn2[360–448] induces an elongated and entangled mitochondrial phenotype. Clone 9 cells expressing GFP in the mitochondrial matrix were transfected with Myc-Mfn2-HR1 (A–A”) or Myc-Mfn2[360–448] (B–B”), and immunostained for the Myc-epitope. Both overexpressed domains distribute to the cytosol due to the lack of membrane binding information (A, B). Mitochondria in cells overexpressing these Mfn2 domains were overly elongated and collapsed (A' and B') compared to those in untransfected control cells (C). Scale bar: 10 µm. (D) Untransfected Clone 9 cells or cells transfected with Mfn2-HR1 or Mfn2[360–448] were assessed for mitochondrial morphology. Approximately 85% and 75% of cells overexpressing Mfn2-HR1 and Mfn2[360–448], respectively, displayed elongated and collapsed mitochondrial phenotype whereas the same phenotype was observed in about 10% of untransfected cells. Error bars represent SEM.

The HR2/HR2 interaction of Mfn molecules has been shown to mediate tethering of opposing mitochondria prior to the membrane fusion [11]. Based on our morphological data using the HR1 mutations, the HR1/HR2 association sequesters HR2 away from the HR2/HR2 interaction and prevents mitochondrial tethering and subsequent fusion. In this context, it is expected that overexpression of the HR1 segment alone would inhibit mitochondrial fusion by binding to HR2 of endogenous Mfn2, thereby resulting in fusion-defective, shorter and more fragmented mitochondrial morphology. However, we found that few cells transfected with Mfn2-HR1 contained fragmented mitochondria, suggesting that the HR2 region of endogenous Mfn2 may not be readily accessible for overexpressed HR1. In addition, the overexpressed HR1 segment showed a diffuse distribution in the cytosol without showing specific localizations (Fig. 5A). Interestingly, many cells overexpressing Mfn2-HR1 contained more elongated and entangled mitochondrial tubules compared to those in untransfected control cells (Fig. 5A', C). The similar phenotype was observed in cells overexpressing Mfn2[360–448] (Fig. 5B'). Cell counting indicated that more than half of the cells transfected with Mfn2-HR1 or Mfn2[360–448] showed an elongated mitochondrial phenotype (Fig. 5D). This observation suggests that HR1 may bind to unknown cytosolic factors and affect mitochondrial fusion/fission.

### Mfn2 interacts with the fission protein DLP1

**Figure 6:**
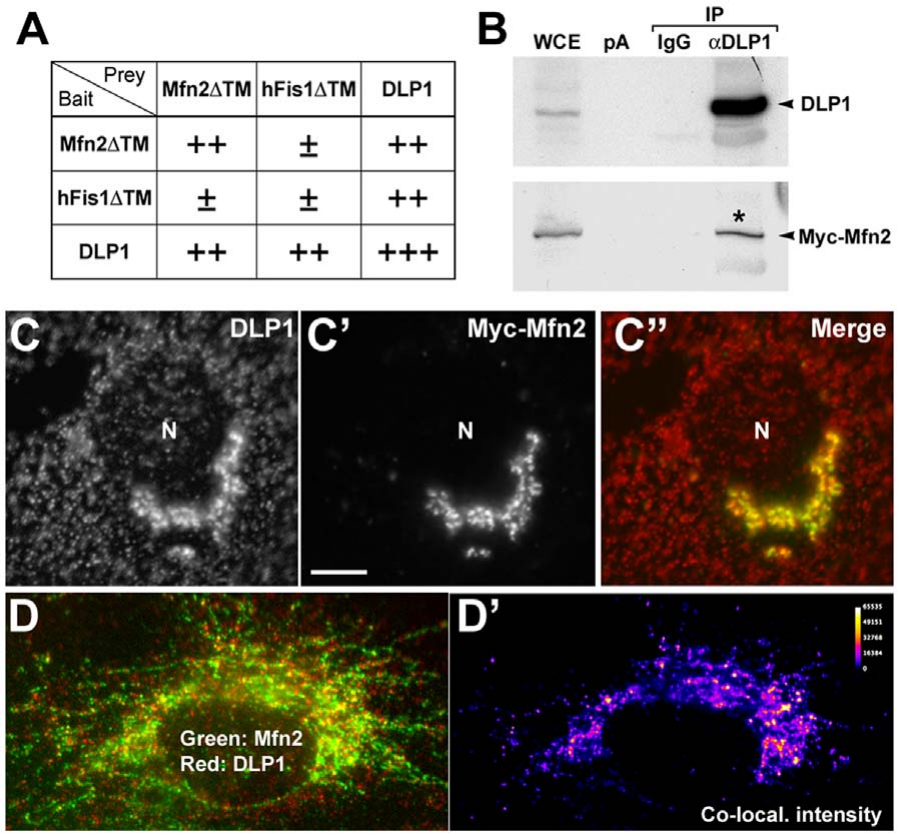
Mfn2 interacts with DLP1. (A) Semi-quantitative Y2H assays show that Mfn2 interacts with DLP1 but not with hFis1. The transmembrane domain of Fis1 was deleted for the Y2H analyses. (B) Co-immunoprecipitation of Mfn2 and DLP1. BHK-21 cells transfected with Myc-Mfn2 were subjected to immunoprecipitation using anti-DLP1 antibodies. Anti-Myc immunoblotting detected the presence of Myc-Mfn2 in the DLP1-immune complex (asterisk) but not in the control (IgG, pA). WCE: whole cell extract. pA: protein A beads. (C–D') Immunofluorescence staining shows co-localization of Mfn2 and DLP1. Clone 9 cells transfected with Myc-Mfn2 were co-stained with anti-DLP1 (C) and anti-Myc (C') antibodies. DLP1 is enriched in the clustered mitochondria where it co-localizes with Mfn2 (C”). In addition, some of the individual DLP1 puncta are co-localized with punctate Myc-Mfn2 in cells containing tubular mitochondria (D). ImageJ Intensity Correlation Analysis for assessing the co-localization (D'). Scale bar: 10 µm. N: nucleus.

Overexpression of full length Mfn2 induces mitochondrial elongation by increasing fusion (Fig. 3D). However, it is unlikely that the HR1 segment lacking the GTPase domain directly increases mitochondrial fusion by binding to endogenous Mfn2. It is possible that overexpressed HR1 in the cytosol sequesters a fusion inhibitory factor such as Mfn-binding protein (MIB) that has been suggested to negatively regulate mitochondrial fusion [38]. However, the region of the Mfn molecule binding to the MIB is not known. Another possibility is that the elongated mitochondrial phenotype in cells overexpressing Mfn2-HR1 or Mfn2[360–448] is due to decreased fission instead of increased fusion. This raises the possibility that the overexpressed HR1 may interfere with mitochondrial fission by interacting with the fission machinery. We therefore tested a potential interaction of Mfn2 with fission proteins, DLP1 and Fis1. The Y2H analyses indicated that there is a significant interaction between Mfn2 and DLP1, whereas an interaction between Mfn2 and Fis1 was not detected (Fig. 6A). The DLP1/DLP1 interaction was also evident as reported previously [33]. Immunoprecipitation experiments showed that a small amount of Mfn2 was co-precipitated with DLP1 (Fig. 6B), suggesting that these two proteins interact with each other probably in a transient manner.

Endogenous DLP1 distributes throughout the cytoplasm and a sub-population is associated with mitochondria [3], [5]. On the other hand, it has been shown that overexpressed Mfn2 predominantly localizes to elongated or clustered mitochondria [36]. Immunofluorescence staining of cells transfected with full length Myc-Mfn2 revealed clear enrichment of DLP1 in the clustered mitochondria induced by Mfn2 overexpression (Fig. 6C–C”), indicating that DLP1 co-localized with Mfn2 in the clustered mitochondria. The significant co-localization of DLP1 with Mfn2 was also observed in elongated tubular mitochondria in cells transfected with Mfn2, showing approximately 13% co-localization frequency of the total pixels (Fig. 6D, D'). Because Mfn2 resides in the mitochondrial outer membrane whereas DLP1 shuttles between the cytosol and mitochondria, it is presumed that the Mfn2/DLP1 interaction that we detected occurs at the mitochondrial surface.

**Figure 7:**
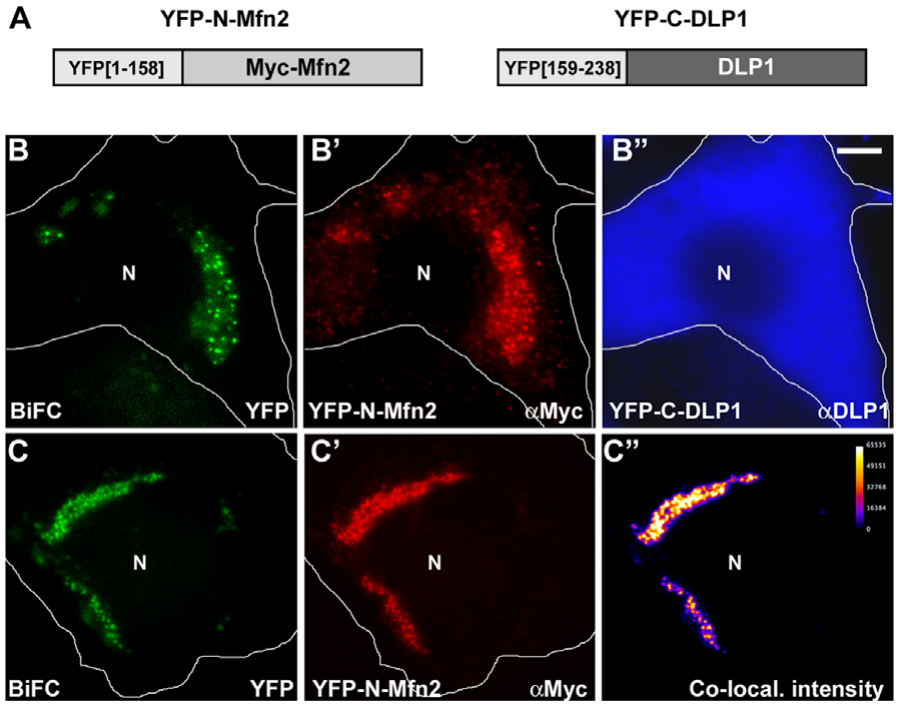
BiFC demonstrating the Mfn2/DLP1 interaction in cells. (A) Constructs used for the BiFC detection of the Mfn2/DLP1 interaction. The YFP N-terminal fragment (YFP[1–158]) was fused to Myc-Mfn2 (YFP-N-Mfn2), and the YFP C-terminal fragment (YFP[159–238]) to HA-DLP1 (YFP-C-DLP1). (B–C”) BHK-21 cells were co-transfected with YFP-N-Myc-Mfn2 and YFP-C-HA-DLP1 and immunostained for Myc (B', C') and DLP1 (B”). Mitochondrial clusters of co-transfected cells show bright punctate YFP signal (B, C). White lines demarcate the cell boundary. The ImageJ Intensity Correlation Analysis was used for assessing co-localization (C”). N: nucleus. Scale bars: 10 µm.

We further tested Mfn2/DLP1 interaction in cells by bimolecular fluorescence complementation (BiFC) in cells. BiFC detects the fluorescent signal produced by two non-fluorescent fragments of yellow fluorescent protein (YFP) when they are brought together by interactions between two proteins fused to each YFP fragment [39], [40]. When G protein β and γ subunits fused to N- and C-terminal fragments of YFP were used as a positive control, we observed the cytoplasmic YFP fluorescence that was more pronounced at the plasma membrane resulting from the Gβ and Gγ interaction, indicating that the BiFC technique is suitable for verifying protein interactions in cytoplasmic environment (Supplemental Fig. S3A, B). Next, we fused Mfn2 to the N-terminal fragment of YFP (YFP[1–158], YFP-N-Mfn2) and DLP1 to the C-terminal fragment of YFP (YFP[159–238], YFP-C-DLP1), and tested for BiFC by co-transfection (Fig. 7A). Many of the transfected cells contained clustered mitochondria caused by Mfn2 overexpression as reported previously [25], [36], [37]. While overexpressed YFP-C-DLP1 filled the cytoplasm (Fig. 7B”), we observed a clear YFP fluorescence in clustered mitochondria, demonstrating DLP1/Mfn2 interaction (Fig. 7BC). The YFP signal was punctate within the mitochondrial clusters where it co-localized with Mfn2, indicating that Mfn2 and DLP1 interact with each other at specific sites of the mitochondrial surface. While BiFC YFP was also observed in tubular mitochondria, it was difficult to obtain these images due to weak signal and rapid bleaching. Negative controls including single transfection of YFP-N-Mfn2 or co-transfection of YFP-N-Mfn2 and YFP-C-Gγ did not show any BiFC YFP (results not shown). These results indicate that the DLP1/Mfn2 interaction we detected is a bona fide cellular event that takes place in the cytoplasm.

### Role of DLP1 in mitochondria fusion

**Figure 8:**
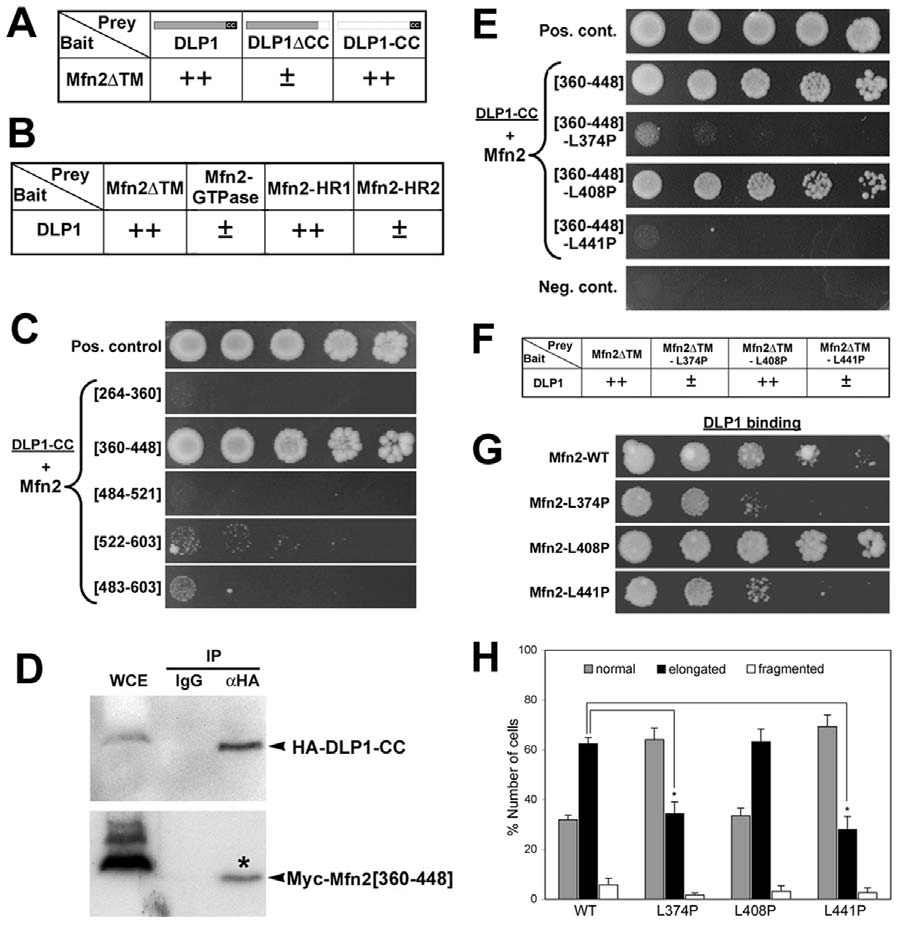
Identification of binding regions in DLP1 and Mfn2, and differential binding of the three HR1 mutants to DLP1. Y2H analyses show that Mfn2ΔTM and DLP1 bind to DLP1-CC (A) and Mfn2-HR1 (B), respectively. (C) Dilution series Y2H analyses show that DLP1-CC binds to Mfn2[360–448]. (D) Co-immunoprecipitation of DLP1-CC and Mfn2[360–448]. Whole cell extract (WCE) from BHK-21 cells co-transfected with HA-tagged DLP1-CC and Myc-tagged Mfn2[360–448] was subjected to anti-HA immunoprecipitation. Myc-Mfn2[360–448] was co-precipitated (asterisk) with HA-DLP1-CC. (E-G) Y2H analyses for interactions of the HR1 mutants with DLP1 using binding segments (E) and full-length proteins (semi-quantitative: F and dilution series: G). L374P and L441P disrupted the DLP1 interactions whereas the L408P mutation maintains this interaction (E). (H) Clone 9 cells expressing mitochondrial GFP were transfected with Mfn2[360–448]-WT, L374P, L408P, or L441P and mitochondrial morphology was assessed for the elongated phenotype. Overexpression of WT and L408P induced mitochondrial elongation whereas significantly decreased elongation was observed with L374P and L441P mutations. Error bars represent SEM.

Further Y2H analyses revealed that Mfn2 interacts with the C-terminal coiled coil domain of DLP1 (DLP1-CC) (Fig. 8A). Analyses using different Mfn2 domains showed that DLP1 interacts with Mfn2-HR1, but not with Mfn2-GTPase or HR2 (Fig. 8B). Because both HR1 and HR2 contain coiled coil motifs but only the HR1 interacts with DLP1, DLP1-CC interacts specifically with Mfn2-HR1 rather than through a nonspecific interaction between two coiled-coil regions. We narrowed down the Mfn2-HR1 region that interacts with DLP1-CC to the Mfn2[360–448] (Fig. 8C), which is the same region that interacts with Mfn2-HR2. Co-immunoprecipitation verified the interaction between DLP1-CC and Mfn2[360-448] (Fig. 8D).

Because Mfn2[360–448] interacts with DLP1, we tested the effect of the three HR1 mutations on the DLP1 binding. Y2H analyses revealed that L374P and L441P mutations abolished the DLP1 binding whereas the L408P mutation did not affect the DLP1 interaction (Fig. 8E). Additionally, we made these three mutations in the full-length Mfn2 and tested their interactions with the full-length DLP1. Both semi-quantitative and dilution series Y2H analyses indicated that the full-length Mfn2-L374P and Mfn2-L441P mutants have markedly reduced interactions with DLP1 whereas the Mfn2-L408P mutant maintains the DLP1 interaction (Fig. 8F,G), consistent with the results obtained with the HR1[360–448] domain. These data along with the HR1/HR2 interaction results (Fig. 3B) indicate that the three HR1 mutations have differential interactions with DLP1 and Mfn2-HR2: the L408P mutation abolished the interaction with Mfn2-HR2 but not with DLP1-CC, whereas the L441P mutation disrupted the HR1/DLP1-CC interaction while maintaining the HR1/HR2 interaction. The L374P mutation disrupts both Mfn2-HR2 and DLP1-CC interactions at the Mfn2-HR1. Additionally, we tested the HR1 mutant segments for their abilities to induce mitochondrial elongation. We found that Mfn2[360–448]-L374P and L441P failed to induce the elongated mitochondrial phenotype whereas L408P mutant was able to promote mitochondrial elongation (Fig. 8H). These morphological data further indicate that L374P and L441P mutations decrease the DLP1 interaction, and support the notion that DLP1 interacts with the Mfn2 HR1 region to induce mitochondrial elongation.

As mentioned earlier, the HR1/HR2 interaction is not necessary for mitochondrial fusion because disruption of this interaction by L374P or L408P mutation does not affect the fusion capacity of Mfn2 (Fig. 3 and 4). On the other hand, the Mfn2-L441P mutation that selectively abolishes the Mfn2/DLP1 interaction does not restore tubular mitochondria in Mfn DKO cells (Fig. 4B, F–F”), suggesting that the Mfn2/DLP1 interaction plays a role in mitochondrial fusion. Because Mfn2-L441P maintains the HR1/HR2 interaction, this result supports the notion that the HR1/HR2 interaction is inhibitory of mitochondrial fusion. Interestingly, although both L374P and L441P mutations disrupted the Mfn2/DLP1 interaction, Mfn2-L374P is capable of mediating fusion (see Discussion).

**Figure 9:**
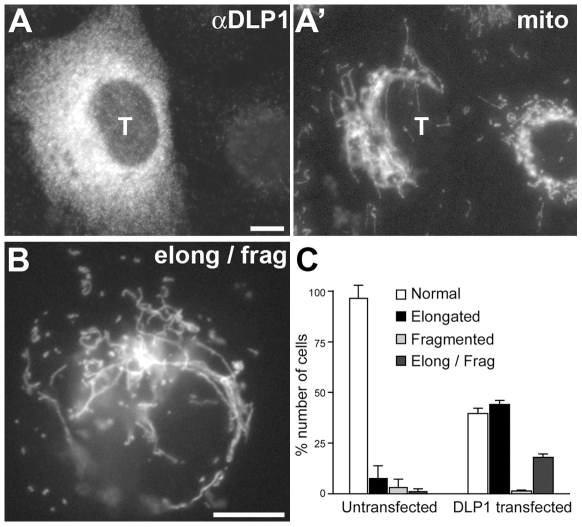
Overexpression of DLP1 induces mitochondrial elongation. Clone 9 cells expressing GFP in the mitochondrial matrix were transfected with untagged full-length wild type DLP1, and immunostained for DLP1. Overexpression of DLP1 induces the formation of elongated and entangled mitochondria (A, A'). A DLP1-overexpressing cell shown in (B) contains long tubular mitochondria along with short fragmented ones. Cell counting indicates that DLP1 overexpression increased mitochondrial elongation (C). Error bars represent SEM. ‘T’ denotes transfected cells. Scale bars: 10 µm.

**Figure 10:**
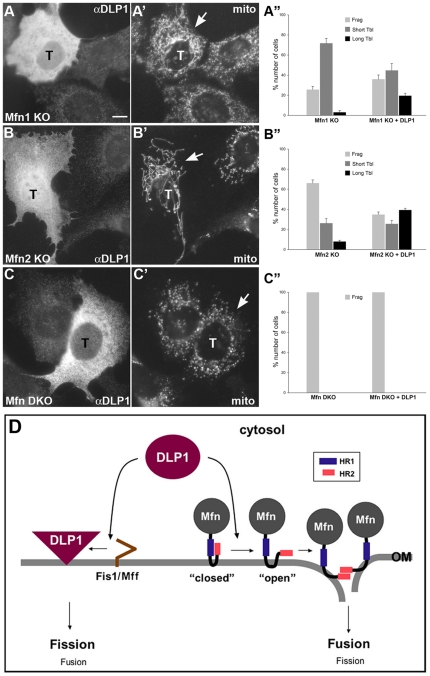
Role of DLP1 in mitochondrial fusion. Mfn1 and 2 single KO cells (mouse embryonic fibroblasts) were transfected with wild type DLP1 and immunostained for DLP1 and cytochrome *c* for mitochondrial morphology. Mitochondria became longer in Mfn1 KO (A–A”) and Mfn2 KO cells (B–B”) overexpressing DLP1 (arrows), whereas those in adjacent untransfected cells were short and fragmented. More marked elongation of mitochondria is apparent in Mfn2 KO cells overexpressing DLP1 (B'). Mfn DKO cells showed completely fragmented mitochondria with DLP1 overexpression (C–C”). Error bars represent SEM. ‘T’ denotes transfected cells. Scale bars: 10 µm. (D) A schematic model describing the regulatory function of DLP1 in mitochondrial fusion and fission. Fission/fusion machineries are in close proximity in the mitochondrial membrane. The HR1/HR2 interaction keeps Mfn2 molecules in the “closed” conformation. The closed conformation could be formed by intra- or inter-molecular HR1/HR2 interaction (Supplemental Fig. S1). Although only the monomeric Mfn2 is shown here, Mfn2 possibly works in a multimeric conformation for the fusion reaction. DLP1 displaces the HR2 at the HR1 to induce the “open” conformation of Mfn2, which allows the HR2 to associate with the HR2 in opposing mitochondria for tethering and outer membrane fusion. During this process, DLP1 becomes locally and temporarily unavailable for Fis1, effectively suppressing the fission process. In the same way, the efficient fission occurs when DLP1 interacts with Fis1.

Protein interactions and morphological data using Mfn2-HR1 mutations suggest that DLP1 may participate in mitochondrial fusion by interacting with Mfn2. In order to further elaborate the role of DLP1 in the fusion process, we examined mitochondrial morphology in cells overexpressing wild type DLP1. If DLP1 were solely a fission protein, overexpressed DLP1 would increase fission, resulting in accumulation of small fragmented mitochondria. However, DLP1-overexpressing cells still contained mostly tubular mitochondria, and cells containing fragmented mitochondria were rarely observed (Fig. 9C) [3], [5]. Instead, we frequently observed more elongated mitochondrial tubules in these cells (Fig. 9A', C). Approximately 45% of the DLP1 transfected cells showed mitochondrial elongation. In addition, we observed long tubules mixed with small fragmented mitochondria in about 20% of the DLP1-overexpressing cells (Fig. 9B, C). This mitochondrial phenotype suggests that increased number of DLP1 molecules enhanced fusion in some tubules while participating in fission of others within the same cell. We obtained more direct evidence for DLP1 in mitochondrial fusion by examining mitochondrial morphology in Mfn single knockout (KO) cells overexpressing DLP1. Mitochondrial fusion still occurs in Mfn1 and Mfn2 single KO cells that contain one Mfn isoform [41]. Both Mfn single KO cell lines contain fragmented and very short tubules caused by a reduction in fusion events [26], [41]. We found that expression of DLP1 in these Mfn single KO cells restores longer tubular mitochondria, supporting the notion that DLP1 increases mitochondrial fusion in these cells (Fig. 10A–B”). Approximately 20% of Mfn1 KO cells transfected with DLP1 showed tubular mitochondria (Fig. 10A”). Surprisingly, the number of cells containing longer mitochondrial tubules increased to 40% in Mfn2 KO cells with DLP1 overexpression (Fig. 10B”). In addition, mitochondria in DLP1-overexpressing Mfn2 KO cells displayed a more pronounced elongation (Fig. 10B'). Although the current study has shown the interaction between DLP1 and Mfn2, these data suggest that DLP1 may interact with both Mfn isoforms to facilitate mitochondrial fusion (see Discussion). Mfn DKO cells showed no tubular mitochondria with DLP1 overexpression due to the complete absence of Mfn molecules (Fig. 10C–C”).

Overall, in this study, we showed an interaction between two heptad repeat regions of Mfn2-HR1 and HR2, and this interaction plays an inhibitory role in mitochondrial fusion. In addition, we found that the Mfn2-HR1 can also interact with the fission protein DLP1, which suggests that the fission protein DLP1 has an additional function in the fusion process through this interaction.

## Discussion

Mfn2 is a multi-domain protein that mediates mitochondrial fusion. Although the presence of domain interactions has been suggested for Mfn proteins [34], [37], no report has definitively shown the HR1/HR2 interaction. A previous study indicated that the Mfn2 lacking the TM and HR2 (GTPase-HR1) localized to the mitochondria upon co-expression with the TM-HR2, suggesting a potential HR1/HR2 interaction of Mfn2 [37]. In the current study, we demonstrated that HR1 interacts directly with the HR2 domain. The overexpressed HR1 domain remained in the cytosol, suggesting that the HR2 domain of endogenous Mfn2 is not readily available for the HR1 interaction. We found that the HR1 domain in the cytosol was unable to interfere with mitochondrial fusion and instead induced elongated mitochondrial morphology, which led to identifying the interaction of Mfn2 with the fission protein DLP1.

Our results demonstrated that Mfn2-HR1 interacts with not only Mfn2-HR2 but also DLP1 at the mitochondrial surface. Three point mutations in the Mfn2[360–448] revealed altered interactions with Mfn2-HR2 and DLP1-CC. Although the detailed structure of this region is undefined, it is possible that three α-helices in this region form a helix bundle that presents binding sites for Mfn2-HR2 and DLP1-CC. Because the mutation in the first helix disrupted both interactions, it is possible that two binding sites share the first helix. Due to small surface areas and an overlap of the two binding sites, HR2 and DLP1-CC likely compete for the HR1 binding, suggesting that binding affinities and local concentrations of these binding domains play a role in differential interactions. It is possible that HR1 is associated with HR2 in resting conditions owing to their close proximity and that their dissociation may be accompanied by the DLP1-CC interaction. Further detailed biochemical and structural analyses will be necessary to define the interaction characteristics of these binding domains.

It is interesting that selective disruptions of HR1/HR2 and Mfn2/DLP1 interactions cause the opposite effects on mitochondrial morphology. Then, what would be the roles of the HR1/HR2 and Mfn2/DLP1 interactions in mitochondrial fission and fusion? It has been shown that mitochondrial fusion requires tethering of opposing mitochondria mediated by the HR2/HR2 interaction of Mfn molecules [11]. In resting conditions, Mfn2-HR1 may be associated with HR2, which we refer to as the “closed” conformation (Fig. 10D). Once HR2 is dissociated from HR1 and becomes free (the “open” conformation), HR2 mediates tethering and mitochondrial fusion can ensue. We speculate that attaining the open conformation of Mfn2 proceeds to mitochondrial fusion, and that the DLP1-CC interaction at the Mfn2-HR1 is one of the mechanisms facilitating this process. It is possible that interactions of HR2 and DLP1-CC at the HR1 are mutually exclusive due to the overlapping binding sites. Therefore, the DLP1-CC interaction at the HR1 would free up HR2 for tethering and fusion (Fig. 10D). This model also explains the fusion competence of Mfn2-L374P despite the abolition of DLP1 interaction by this mutation. Because the HR1/HR2 interaction is also absent in Mfn2-L374P, this mutant takes the open conformation and is able to mediate fusion.

An open question is how critical the Mfn2/DLP1 interaction is in mitochondrial fusion. It is clear that the dominant role of DLP1 is in the fission process. Cells treated with DLP1 siRNA or DLP1 knockout cells show elongated mitochondrial tubules [42], [43], indicating a decrease of fission, not fusion. However, a small change of the overall fission/fusion balance can result in a drastic alteration in the terminal mitochondrial morphology [41], [44]. Although the overly elongated mitochondria in DLP1-knockout or knockdown cells are clearly a fission-defective phenotype, the extent that the lack of DLP1 decrease mitochondrial fusion in these cells is unknown. Possibly, DLP1-knockout/knockdown decreases fission more than fusion, inducing elongated mitochondrial phenotype. Additionally, the notion that fusion is affected to a lesser extent compared to fission upon DLP1 knockout/knockdown suggests that the DLP1 binding to HR1 is not the sole mechanism to dissociate the HR1/HR2 interaction promoting mitochondrial fusion.

Intriguing morphological changes brought about by overexpression of wild type DLP1 indicates that DLP1 can act as a fusion factor. Contrary to the idea that mitochondrial fission should increase upon overexpressing DLP1, fragmented mitochondria were rarely observed in DLP1-overexpressing cells as shown before [3], [5]. While the absence of mitochondrial fragmentation in these cells is possibly due to the limited number of the putative DLP1 receptor Fis1 or Mff, the most noticeable mitochondrial morphology in DLP1-overexpressing cells was elongated tubules that were often collapsed around the nucleus (Fig. 9A'). This mitochondrial phenotype suggests that excess DLP1 that does not engage in fission (limited by the number of Fis1/Mff molecules) may increase fusion by interacting with Mfn2. By overexpressing DLP1 in Mfn single KO cells, more direct evidence for the role of DLP1 in mitochondrial fusion was obtained (Fig. 10). Restoring long tubular mitochondria by increasing the number of DLP1 molecules in Mfn single KO cells containing residual fusion activity provides strong evidence for the role of DLP1 in facilitating fusion. Another interesting finding was that DLP1 could also interact with the Mfn1 isoform, as DLP1 overexpression in Mfn2 KO cells form markedly elongated mitochondria (Fig. 10B'). Mfn1 and Mfn2 are highly similar to each other in their domain structures, and both homomeric and heteromeric complexes mediate mitochondrial fusion [12], [26], [45]. Mfn1 has been shown to be more ubiquitously expressed throughout mammalian tissues as well as in fibroblasts and cell lines whereas Mfn2 are more tissue specific showing high abundance in the heart [27]. The more pronounced effect of mitochondrial elongation in Mfn2 KO cells containing only the Mfn1 isoform suggests that DLP1 interacts with Mfn1 more efficiently or that Mfn1 may be more abundant than Mfn2 in this fibroblast cell line. Further studies are necessary to verify whether Mfn1 has similar interaction characteristics to Mfn2 intra-molecularly and with DLP1. Regardless, the mitochondrial phenotype induced by DLP1 overexpression supports the notion that DLP1 can participate in the fusion process in addition to its function as the fission protein.

An extended scenario based on this model is that DLP1 can be a regulatory factor determining fission or fusion by binding differentially to Fis1/Mff and Mfn2. DLP1 has been shown to be in close proximity to Mfn2 [46], indicating the presence of a fission/fusion microenvironment at the mitochondrial surface. It is possible that fission and fusion machineries stay close together on the mitochondrial membrane, poised to execute either fission or fusion upon specific cellular cues. For efficient execution of fission or fusion, it would be necessary to activate the one process while concomitantly suppressing the other. Interaction of DLP1 with Mfn2 would allow mitochondrial fusion as discussed above. At the same moment, the DLP1/Mfn2 interaction would decrease the DLP1 availability for Fis1/Mff within the fission/fusion microenvironment, thereby efficiently shifting the balance to the fusion process (Fig. 10D). In the same way, an efficient shift to fission would occur when DLP1 interacts with Fis1/Mff. A similar mechanism has been reported for the membrane fission dynamin Vps1p participating in yeast vacuole fusion, in which a reversible interaction of Vps1p with a fusion t-SNARE protein promotes membrane fusion with a concomitant inhibition of fission [35].

In this study, we identified previously unrecognized interactions involving fusion and fission proteins, Mfn2 and DLP1. Our results show that Mfn2-HR1 interacts with HR2 and DLP1-CC, and that these interactions regulate mitochondrial morphology. Many questions still remain, including the mechanisms regulating the differential interactions, multimeric states of Mfn2/DLP1/Fis1 involved in these interactions, and Mfn isoform specificity for these molecular interactions. Several phosphorylation events have been suggested within the binding regions of Mfn2 and DLP1, most notably the serine 442 in the Mfn2-HR1 [23], [47]–[50]. Differential phosphorylation within the binding domains of Mfn2 and DLP1 in response to different cellular signals may regulate these interactions to control mitochondrial morphology. While further study is necessary, the current study presents the first evidence for the interaction between mitochondrial fission and fusion proteins, and provides a novel insight as to how the physical and functional communications of fission and fusion machineries at the fission/fusion site regulate mitochondrial morphology.

## Materials and Methods

### Cell lines and transfection

Clone 9 (ATCC CRL-1439) and BHK-21 cells (ATCC CCL-10) were maintained in Ham's F-12K and Dulbecco's modified Eagle's media, respectively. Mfn1 and Mfn2 single and double knockout mouse embryonic fibroblasts were obtained from Dr. David Chan (California Institute of Technology). Cell culture media were supplemented with 10% fetal bovine serum, nonessential amino acids, L-glutamine and penicillin/streptomycin. Clone 9 cells stably expressing mitochondrial matrix-targeted GFP were maintained in 200 µg/ml G418 [8]. Transfection was performed using lipofectamine (Invitrogen, Inc.) according to the manufacturer's instruction.

### Plasmid construction

Standard PCR-based amplification and subcloning techniques were used for plasmid construction. Correct sequences of subcloned DNA were verified by DNA sequencing. For yeast two-hybrid constructs, DNA fragments encoding indicated Mfn2, DLP1, and hFis1 regions were cloned into yeast pGADT7 and pGBKT7 vectors that contain *LEU2* and *TRP1*, respectively, as nutritional selection markers (Clontech). The mammalian expression vector pcDNA3 (Invitrogen) was used as a backbone plasmid for all the constructs used for mammalian cell transfection.

### Yeast two-hybrid procedure

Matchmaker™ Two-Hybrid System 3 (Clontech) was used for two-hybrid studies. Standard yeast genetic techniques and media were used. SD (synthetic dropout) and YPD media supplemented with 2% dextrose were prepared as described previously [51]. AH109 (*MATa, trp1-901, leu2-3, 112, ura3-52, his3-200, gal4Δ, gal80Δ, LYS2 : : GAL1_UAS_-GAL1_TATA_-HIS3, GAL2_UAS_-GAL2_TATA_-ADE2, URA3 : : MEL1_UAS_-MEL1_TATA_-lacZ*) was used as yeast host strain. Lithium acetate-mediated yeast transformation was used according to Yeast Protocols Handbook (Clontech User Manual). Western blot analysis was used to confirm the expression of the proper constructs in host strain after transformation. Interactions between bait and prey proteins were evaluated by growth in SD/-Leu/-Trp/-His. Briefly, yeast transformants grown in the SD/-Leu/-Trp liquid media was collected and resuspended at a density of 1.0 OD unit/ml at 600 nm. For semi-quantitative analyses, 50 µl of the 1∶625 dilution was spread onto both SD/-Leu/-Trp and SD/-Leu/-Trp/-His plates, and ratios of the colony numbers on the SD/-Leu/-Trp/-His plate and the SD/-Leu/-Trp plate were used for indications of the interaction strength. For serial dilution assays, the 1:5 serial dilutions were spotted onto the SD/-Leu/-Trp/-His plate and grown for four days for visual assessment of interactions.

### Immunoprecipitations

Appropriate DNA constructs were transfected to BHK-21 cells and expressed for 24 hours. Cell lysates were prepared in PBS containing 1% Triton X-100 and protease inhibitors cocktail (Sigma-Aldrich) and cleared by centrifugation. The supernatant (whole cell extract) was incubated with anti-HA or anti-DLP1 antibodies, and the immune complex was isolated with Protein A-Sepharose (Sigma-Aldrich). Normal rabbit IgG was added in place of antibodies for negative control experiments. Where necessary, whole cell extract was pre-cleared by rabbit IgG and Pansorbin (CalBiochem) before incubating with antibodies. In some experiments, Protein A-conjugated magnetic beads (Invitrogen) were used instead of the Protein A-Sepharose. After washing, bead-bound immune complex was eluted using 200 mM glycine (pH 2.0) for immunoblot analyses.

### Indirect immunofluorescence

Indirect immunofluorescence was performed as previously described [4], [7]. Cells were grown on glass coverslips for 16–24 h before transfection. Cells were fixed with 4% paraformaldehyde (Electron Microscopy Sciences) and permeabilized with 0.1% Triton X-100 (Pierce Biotechnology). After blocking, cells were incubated with primary and then secondary antibodies. Mouse anti-Myc (Sigma-Aldrich), human anti-mitochondria [5], and/or rabbit anti-DLP1 were used as the primary antibody and Alexa 488- or Alexa 594-conjugated anti-mouse, anti-rabbit or anti-human IgG (Molecular Probes Inc.) for secondary antibodies. After rinsing, coverslips were mounted in ProLong antifade reagent (Molecular Probes Inc.) on glass slides. Fluorescence images were acquired with an Evolution QEi camera (Mediacybernetics, Inc.) and analyzed/adjusted using ImageJ (Wayne Rasband, NIH) and Adobe Photoshop (Adobe Systems Inc.) software.

## Supporting Information

Figure S1 **Intra- and inter-molecular interactions of Mfn2 involving HR1 and HR2.** (A) GST-HR2 was incubated with 6xHis-tagged HR1 and HR1-HR2 separately or with the mixture of the two proteins. Bound proteins were pulled down with glutathione beads and analyzed by anti-His immunoblotting. In: input, B: bound. GST-HR2 pulled down HR1 and HR2 in separate incubations. HR2 binds preferentially to HR1 in the incubation with the mixture of HR1 and HR1-HR2. (B) Mfn2-HR1 and HR2 may be associated within the same molecule by an intra-molecular interaction. The anti-parallel HR1/HR2 interaction between two Mfn2 molecules would form the dimeric configuration whereas parallel HR1/HR2 interactions lead to the multimeric configuration.
(TIF)

Figure S2 **Morphometric analyses for mitochondrial shapes induced by Mfn2 mutants.** Form factor (FF) and Aspect ratio (AR) have a minimal value of 1 when it is a small perfect circle and the values increase as mitochondria become elongated. AR is a measure of mitochondrial length, and increase of FF represents increase of mitochondrial length as well as branching. Mitochondria in cells expressing WT, L374P or L408P have increased values of both AR and FF compared to untransfected (UT) cells whereas those of cells expressing L441P have the value close to 1 for small circular morphology. The number of mitochondria increased in the Mfn2-L441P mutant cells, indicating mitochondrial fragmentation, whereas the numbers decreased with expression of the other mutants and wild type for an increased fusion.
(TIF)

Figure S3 **BiFC by interaction of Gβ and Gγ.** (A and B) G protein β and γ subunits fused to N- and C-terminal fragments of YFP as a positive control. Upon co-transfection of positive control plasmids, the cytoplasmic YFP fluorescence with a concentration at the cell cortex was observed, indicating that Gβ and Gγ interact in cells pronouncedly at the plasma membrane.
(TIF)
