# Supplementary figures and images for: Control of Mitochondrial Morphology Through Differential Interactions of Mitochondrial Fusion and Fission Proteins
Source: PLoS One. 2011 May 27;6(5):e20655. doi: 10.1371/journal.pone.0020655 (PMC3103587; doi:10.1371/journal.pone.0020655)

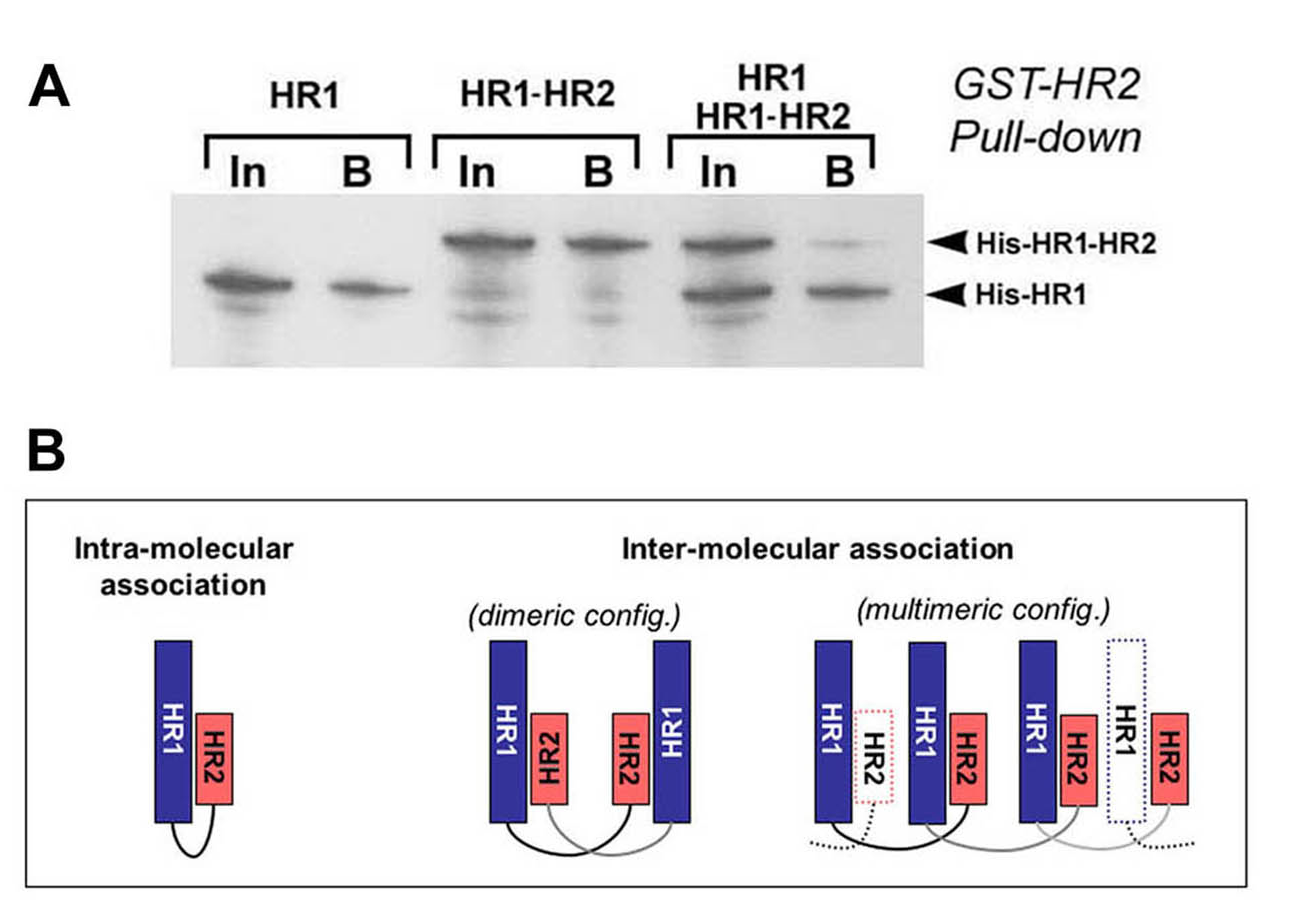

Supplement: Figure S1 — Intra- and inter-molecular interactions of Mfn2 involving HR1 and HR2. (A) GST-HR2 was incubated with 6xHis-tagged HR1 and HR1-HR2 separately or with the mixture of the two proteins. Bound proteins were pulled down with glutathione beads and analyzed by anti-His immunoblotting. In: input, B: bound. GST-HR2 pulled down HR1 and HR2 in separate incubations. HR2 binds preferentially to HR1 in the incubation with the mixture of HR1 and HR1-HR2. (B) Mfn2-HR1 and HR2 may be associated within the same molecule by an intra-molecular interaction. The anti-parallel HR1/HR2 interaction between two Mfn2 molecules would form the dimeric configuration whereas parallel HR1/HR2 interactions lead to the multimeric configuration. (TIF) [file pone.0020655.s001.tif]

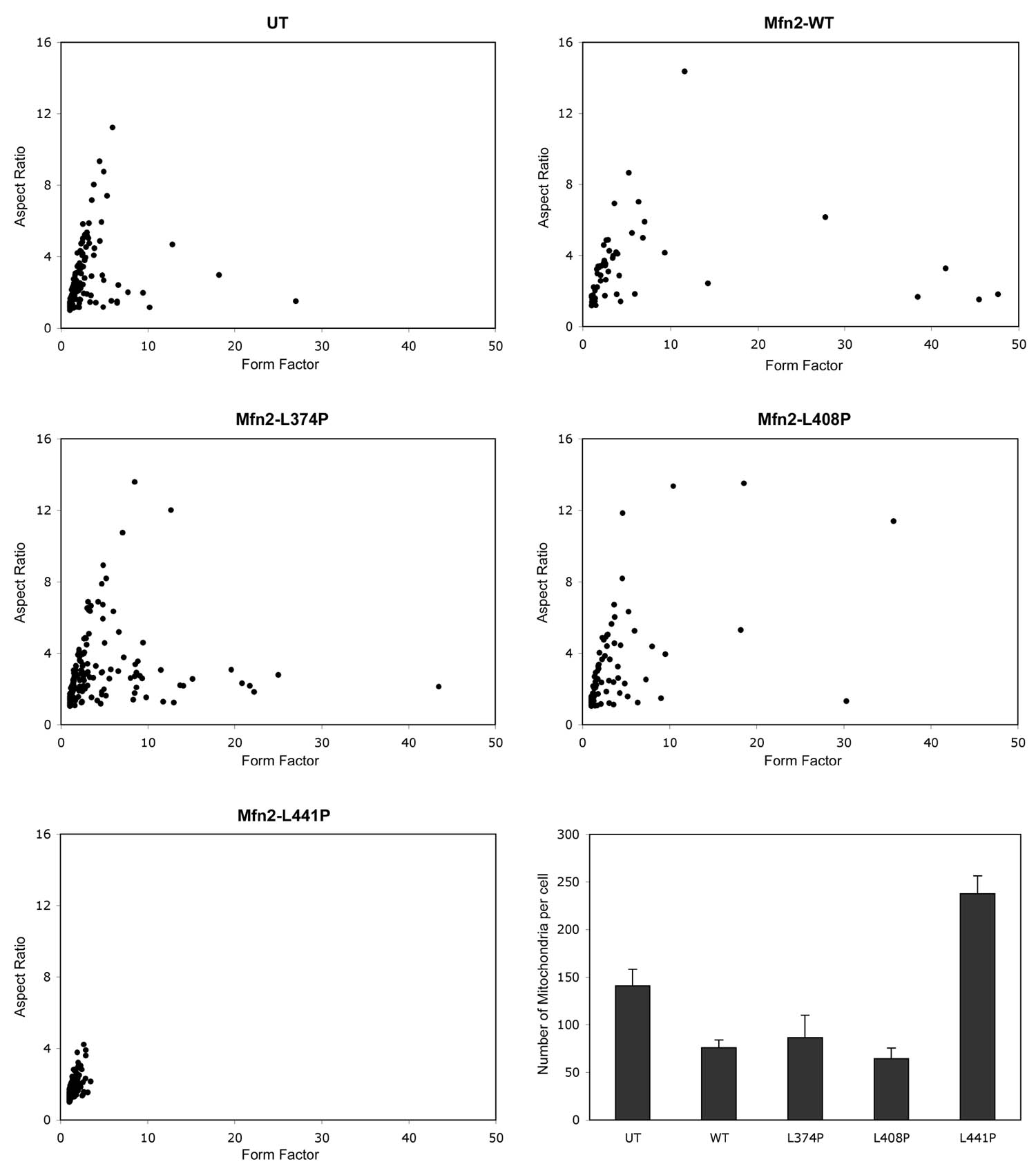

Supplement: Figure S2 — Morphometric analyses for mitochondrial shapes induced by Mfn2 mutants. Form factor (FF) and Aspect ratio (AR) have a minimal value of 1 when it is a small perfect circle and the values increase as mitochondria become elongated. AR is a measure of mitochondrial length, and increase of FF represents increase of mitochondrial length as well as branching. Mitochondria in cells expressing WT, L374P or L408P have increased values of both AR and FF compared to untransfected (UT) cells whereas those of cells expressing L441P have the value close to 1 for small circular morphology. The number of mitochondria increased in the Mfn2-L441P mutant cells, indicating mitochondrial fragmentation, whereas the numbers decreased with expression of the other mutants and wild type for an increased fusion. (TIF) [file pone.0020655.s002.tif]

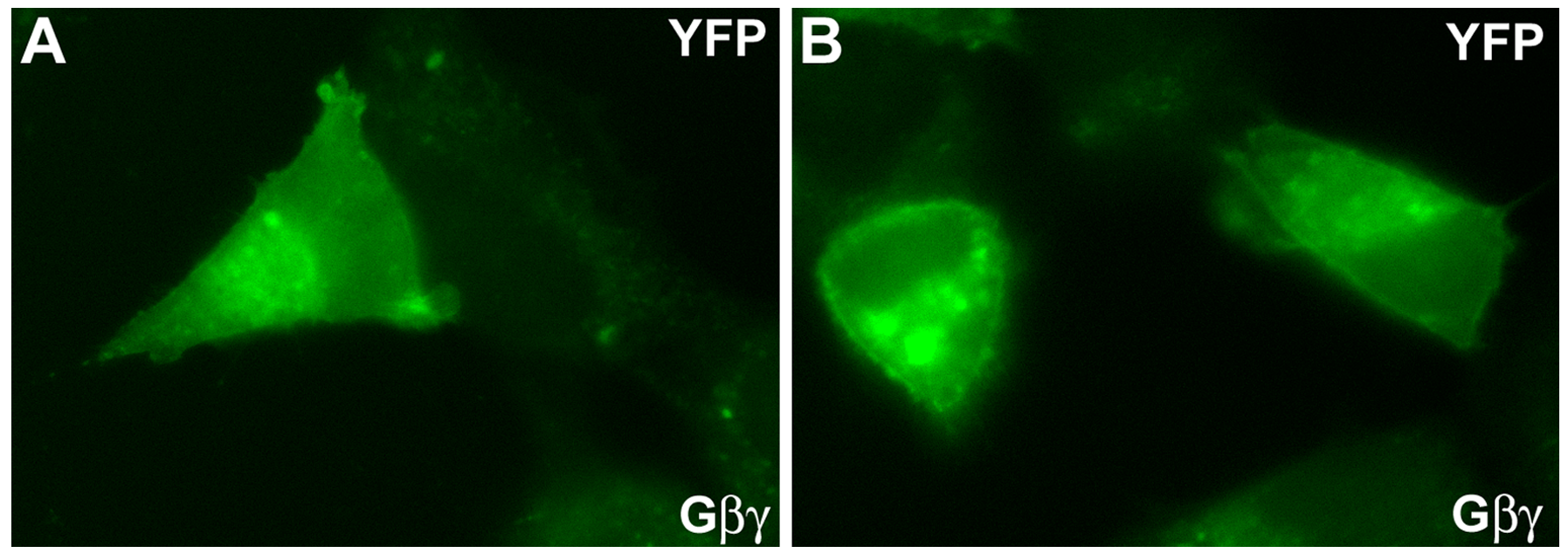

Supplement: Figure S3 — BiFC by interaction of Gβ and Gγ. (A and B) G protein β and γ subunits fused to N- and C-terminal fragments of YFP as a positive control. Upon co-transfection of positive control plasmids, the cytoplasmic YFP fluorescence with a concentration at the cell cortex was observed, indicating that Gβ and Gγ interact in cells pronouncedly at the plasma membrane. (TIF) [file pone.0020655.s003.tif]
